# Retrospective Analysis on Antimicrobial Resistance Trends and Prevalence of β-lactamases in *Escherichia coli* and ESKAPE Pathogens Isolated from Arabian Patients during 2000–2020

**DOI:** 10.3390/microorganisms8101626

**Published:** 2020-10-21

**Authors:** Mahfouz Nasser, Snehal Palwe, Ram Naresh Bhargava, Marc G. J. Feuilloley, Arun S. Kharat

**Affiliations:** 1 Department of Biotechnology, Dr. Babasaheb Ambedkar Marathwada University, Sub-Campus, Osmanabad 413 528, MS, India; mahfouznasser@yahoo.com; 2 National Center for Public Health Laboratories, Hodeidah, Yemen; 3 Department of Environmental Science, S. B. College of Science, Aurangabad 431001, India; snehalpalwe7@gmail.com; 4 Department of Microbiology, Babasaheb Bhimrao Ambedkar University, Lucknow 226025, India; bharagavarnbbau11@gmail.com; 5 Laboratory of Microbiology Signals and Microenvironments, LMSM EA 4312, University of Rouen, Normandy, F-27000 Evreux, France; 6 Laboratory of Applied Microbiology, School of Life Sciences, Jawaharlal Nehru University, New Mehrauli Road, New Delhi 110067, India

**Keywords:** ESBL, *E. coli*, ESKAPE pathogens, antibiotic resistance, β-lactamase, Arab region

## Abstract

The production of diverse and extended spectrum β-lactamases among *Escherichia coli* and ESKAPE pathogens is a growing threat to clinicians and public health. We aim to provide a comprehensive analysis of evolving trends of antimicrobial resistance and β-lactamases among *E. coli* and ESKAPE pathogens (*Enterococcus faecium, Staphylococcus aureus, Klebsiella pneumoniae, Acine to bacter baumannii, Pseudomonas aeruginosa*, and *Enterobacter* species) in the Arabian region. A systematic review was conducted in Medline PubMed on papers published between January 2000 and February 2020 on countries in the Arab region showing different antibiotic resistance among *E. coli* and ESKAPE pathogens. A total of *n* = 119,144 clinical isolates were evaluated for antimicrobial resistance in 19 Arab countries. Among these clinical isolates, 74,039 belonged to *E. coli* and ESKAPE pathogen. Distribution of antibiotic resistance among *E. coli* and ESKAPE pathogens indicated that *E. coli* (*n* = 32,038) was the predominant pathogen followed by *K. pneumoniae* (*n* = 17,128), *P. aeruginosa* (*n* = 11,074), methicillin-resistant *S. aureus* (MRSA, *n* = 4370), *A. baumannii* (*n* = 3485) and *Enterobacter* spp. (*n* = 1574). There were no reports demonstrating *Enterococcus faecium* producing β-lactamase. Analyses revealed 19 out of 22 countries reported occurrence of ESBL (Extended-Spectrum β-Lactamase) producing *E. coli* and ESKAPE pathogens. The present study showed significantly increased resistance rates to various antimicrobial agents over the last 20 years; for instance, cephalosporin resistance increased from 37 to 89.5%, fluoroquinolones from 46.8 to 70.3%, aminoglycosides from 40.2 to 64.4%, mono-bactams from 30.6 to 73.6% and carbapenems from 30.5 to 64.4%. An average of 36.9% of the total isolates were reported to have ESBL phenotype during 2000 to 2020. Molecular analyses showed that among ESBLs and Class A and Class D β-lactamases, *bla*_CTX-M_ and *bla*_OXA_ have higher prevalence rates of 57% and 52.7%, respectively. Among Class B β-lactamases, few incidences of *bla*_VIM_ 27.7% and *bla*_NDM_ 26.3% were encountered in the Arab region. Conclusion: This review highlights a significant increase in resistance to various classes of antibiotics, including cephalosporins, β-lactam and β-lactamase inhibitor combinations, carbapenems, aminoglycosides and quinolones among *E. coli* and ESKAPE pathogens in the Arab region.

## 1. Introduction

Before the discovery of penicillin by Alexander Fleming in 1928, infectious diseases accounted for high morbidity and mortality worldwide [1]. After a decade of antibiotic discovery, they were made available for the effective treatment of various bacterial diseases and although the United States Surgeon General at that time, Dr. Stewart, never stated that it was time to close the book on infections, the fraternity of clinicians started thinking that they had won the battle against infectious diseases [2]. However, the inactivation of these antibiotics by bacterial pathogens due to expression of various enzymatic and non-enzymatic resistance mechanisms started appearing within a few years after their introduction in to the market and their subsequent clinical usage [3]. During 1940–1970, diverse classes of antibiotics inhibiting various essential bacterial targets such as DNA, ribosome, peptidoglycan synthesis were introduced, mainly providing antibacterial activity against Gram-positive pathogens, especially *Staphylococcus aureus* and streptococci [4]. During the same period, β-lactams, aminoglycosides and quinolines were discovered and predominantly used in treatment of infections caused by staphylococci and streptococci.

Among these three classes of antibiotics, β-lactams were the mainstay of therapy owing to their well-established clinical safety and efficacy. However, in early 1975, Neu (1975) documented that infections caused by Gram-negative pathogens had started to become more prevalent over Gram-positive infections, which were predominantly community bacterial infections with plenty of therapeutic options available for treatment [5]. With narrow spectrum activity of these old antibiotics, Gram-negative infections associated with *Escherichia coli*, *Klebsiella pneumoniae* and *Enterobacter* spp. began to rise, and bacterial infections shifted from community to hospitals. However, limited therapeutic options were available for the treatment of these nosocomial infections, compelling clinicians to use available therapeutic options with compromised Gram-negative efficacy. This was the era of selection and dissemination of various β-lactamases including inhibitor resistant β-lactamases in etiologically prevalent organisms such as *E. coli*, *K. pneumoniae* and *Enterobacter*.

On the other hand, the incidence of infections caused by other Gram-negative opportunistic pathogens such as *Pseudomonas aeruginosa* and *Acine to bacter baumannii* in hospital acquired bacterial pneumonia and Ventilator Associated-bacterial Pneumonia (VAP) further complicated the treatment, thus challenging treatment options. Therefore, post 1970, various structural modifications in β-lactam antibiotics led to the discovery of 3rd- and 4th-generation cephalosporins and carbapenems, which possessed broad spectrum activity encompassing these pathogens. These clinically significant pathogens belonged to *E. coli* and ESKAPE pathogens. The ability of *E. coli* and ESKAPE pathogens to acquire and disseminate various resistance genes led to Multiple-Drug Resistance (MDR). There are six nosocomial pathogens in the acronym ESKAPE: *Enterococcus faecium, Staphylococcus aureus, Klebsiella pneumoniae, Acine to bacter baumannii, Pseudomonas aeruginosa* and *Enterobacter* spp. [6]. Globally, nosocomial infections caused by MDR *E. coli* and ESKAPE pathogens are associated with the highest death risk and incur huge treatment costs [7]. Various regulatory agencies such as the US Centers for Disease Control and Prevention (CDC), European Centre for Disease Prevention and Control (ECDC) and the World Health Organization (WHO) have provided detailed information on the antibiotic susceptibility of contemporary susceptible and resistant ESKAPE pathogens based on various surveillance studies. According to a WHO report, at least 700,000 people die each year, and it is estimated that drug-resistance could cause 10 million deaths each year by 2050, with a significant socio-economic impact [8]. Due to globalization, systematic analysis of *E. coli* strains expressing β-lactamases and spread of antibiotic resistance traits among ESKAPE pathogens from both developed and developing countries is of paramount importance.

Such systematic surveillance data is not available for various developing countries such as Africa, the Middle East, and European countries outside the European Union [9]. Only limited data are available for even the Arab regions, which is a geographical term for the countries located between two continents: Asia and Africa. These Arab regions comprise twenty-two countries, twelve of which are located in western Asia, while ten are in northern Africa. Herein, we performed an extensive literature survey covering the period from January 2000 to February 2020 and the antibiotic resistance trends among *E. coli* and ESKAPE pathogens from the 22 countries of the Arab region. Based on the available data, phenotypic and genotypic expression of various β-lactamase traits were highlighted country-wise. As per the methodological standards outlined for the conduct of PRISMA Intervention review, we divided the study period (2000–2020) into four Sub-groups; 2000–2005, 2006–2010, 2011–2015, and 2016–2020 and then compared and analyzed antibiotic resistance trends between these periods.

## 2. Materials and Methods

**Figure 1:**
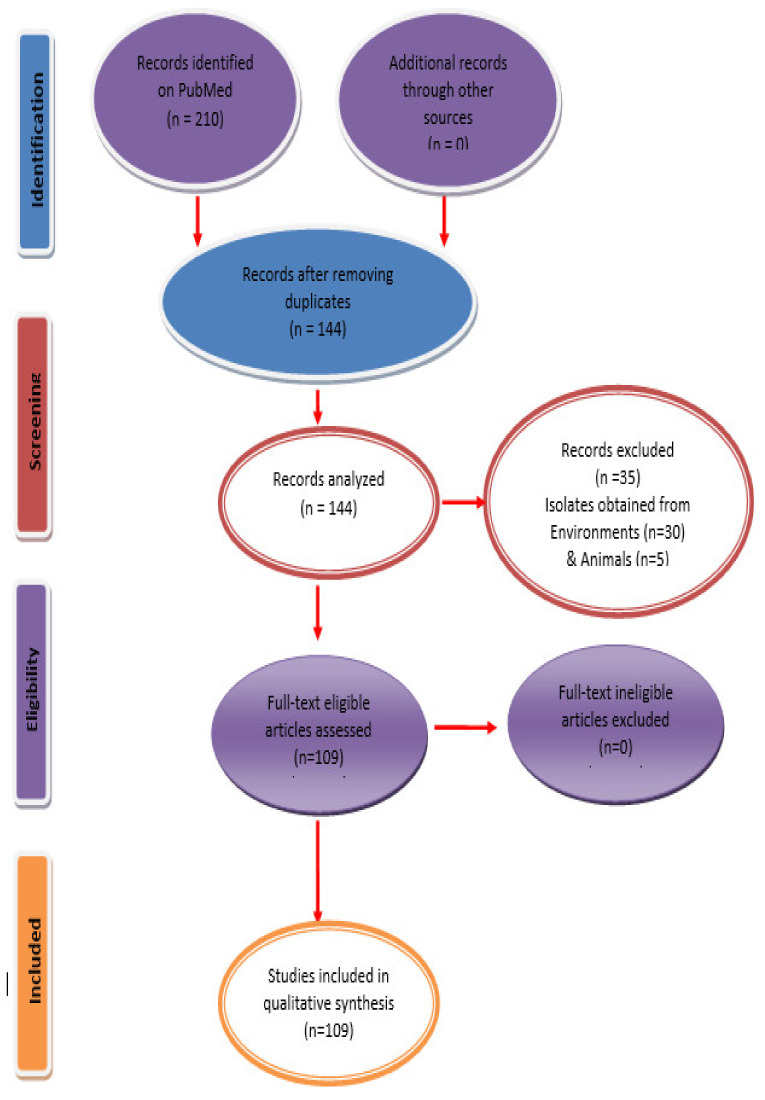
PRISMA flow diagram for study inclusion. PRISMA = preferred reporting items for systematic reviews and meta-analysis.

Various studies showing activity of different classes of antibiotics against *E. coli* and ESKAPE pathogens, published between January 2000 to February 2020 in Medline PubMed databases, were reviewed. We followed the Methodological Standards guidelines for the Conduct of PRIMSA Intervention Review to study the antibiotic resistance trends over 20 years within 19 Arab countries [10] (see Figure 1). We performed a systematic review of the available literature on specific antimicrobial resistance data, primarily for Arab countries for which no systematic surveillance data on antimicrobial resistance data is available. Among *E. coli* and ESKAPE pathogens, this review is mainly focused on *E. coli, K. pneumoniae, P. aeruginosa* and *A. baumannii*, as the resistance data against these pathogens is highly variable worldwide and is gathering massive attention owing to the challenges, severity of infections and treatment threats posed by these pathogens. Out of 22 countries studied in this report, none reported the presence of *Enterococcus faecium* expressing ESBL; hence, this review did not focus on other types of *Enterococci* reported from these countries during period of 2000 through 2020. The search words were “antibiotics resistance”, “Arab countries” and “Gram-negative pathogens”.

Data selection: Inclusion criteria were studies published in English, the date of publication between 1 January 2000 and 29 February 2020, original research, human reports, reports on one or more of ESKAPE Pathogens with or without *E. coli*, and reports on resistance cases indicating β-lactamase.

Data extraction: Relevant data were extracted using a standardized form of collection from published articles. The analyses included: country, year of isolate collection, cultures isolated from various clinical specimens such as intra-abdominal, urinary tract, skin, wound and respiratory infections. The bacterial species analyzed were: *E. coli*, *K. pneumoniae*, *A. baumannii, P. aeruginosa* and *Enterobacter* spp. The number of isolates tested and the antibiotic susceptibility shown here is based on the Clinical Laboratory Standard Institute (CLSI, USA) interpretive criteria. Both phenotypic (disk diffusion methods) and genotypic (PCR technique) data were taken into consideration while estimating the % prevalence of ESBLs.

Statistical analysis: Using SPSS version 26^®^ of statistical software (IBM, USA), data were generated and analyzed. Descriptive statistics were determined using the marginal mean function in SPSS and the calculated mean was estimated.

## 3. Results

Due to globalization, the emergence and spread of antibiotic resistance among organisms causing frequent outbreaks has become prevalent. Nineteen out of twenty-two Arab countries (86.3%) reported the occurrence of antimicrobial resistance either in *E. coli* or ESKAPE pathogens. Similar data were not found for Mauritania, Somalia, and the Comoros Islands. Between January 2000 and February 2020, there were 109 articles published on antibiotic resistance and β-lactamase(s) from 22 Arab countries.

### 3.1. Saudi Arabia

There were a total of thirty single or multiple hospital-based surveillance studies, which evaluated the prevalence of antimicrobial resistance among *E. coli* and ESKAPE pathogens. These studies comprised of (*n* = 52,033) *E. coli* and ESKAPE pathogens of which, *E. coli* were the highest (*n* = 22,493) followed by *K. pneumoniae* (*n* = 11,959), *P. aeruginosa* (*n* = 9854), *S. aureus* (*n* = 3829), *A. baumannii* (*n* = 2582), and *Enterobacter* spp. (*n* = 1316). Most of these isolates were recovered from patients admitted to Intensive Care Units (ICU) and patients with complicated Urinary Tract Infections (cUTI). Studies reported from Saudi Arabia showed significant increased resistance rates to various antimicrobial agents during the years 2000 through 2020. In comparison to β-lactam and beta-lactamase inhibitors (amoxicillin-clavulanic acid and piperacillin-tazobacam) resistance for standalone cephalosporins, such as cefuroxime, cefotaxime, ceftazidime, and cefepime was considerably high. Similarly, increased resistance rates were observed for aminoglycosides (gentamicin and amikacin), and quinolones (ciprofloxacin) compared to relatively lower resistance to carbapenem, imipenem and meropenem, shown in Table 1 [11,12,13,14,15,16,17,18,19,20,21,22,23,24,25,26,27].

Antibiotic resistance trends were also well supported by the identification of β-lactamases in these isolates. Isolates expressing various ESBLs were confirmed by double-disk potentiation method, thirteen studies from Saudi Arabia reported the phenotypic prevalence of ESBLs in *E. coli* and ESKAPE pathogens. These studies showed that during the initial study period of year 2000, ESBL prevalence was relatively low (4.8%), but increased dramatically to 72% by 2020 in these countries based on the data obtained from 93,961 clinical samples [13,14,19,20,22,24,25,28,29,30,31,32,33]. The PCR analysis of various β-lactamases traits shown in Table 2 indicates that the prevalence of *bla*_SHV_, *bla*_TEM_ and *bla*_CTX-M_ among the *E. coli* and ESKAPE pathogens increased from between 2.6–8.5 to 84.1–97.3%, the *bla*_OXA,_ enzyme during this period increased from 26.2% to 85.7%, and increasing resistance for *bla*_PER_ *bla*_GES_ β-lactamases was also demonstrated. On the other hand, *bla*_VIM_ and *bla*_NDM_ b-lactamases were the most prevalent metallo-β-lactamases resistance encoding genes, showing a remarkable increase, along with the *bla*_IMP_ trait [11,12,15,16,17,18,19,20,21,24,25,28,29,33,34,35,36,37].

### 3.2. United Arab Emirates

There were five single or multiple hospital-based surveillance studies demonstrating the prevalence of antimicrobial resistance among *E. coli* and ESKAPE pathogens including 1191 isolates, of which *E. coli* was the highest (*n* = 654), followed by *K. pneumoniae* (*n* = 524), and *Enterobacter* spp. (*n* = 13). These pathogens were mostly isolated from patients admitted to the ICU and also from patients with cUTI and blood samples (septicemia). These studies demonstrated substantially higher rates of resistance to various antimicrobials in the last 20 years, for example, between 2014 and 2016, the resistance to ceftazidime, cefotaxime and ciprofloxacin was 72%, 73% and 74%, respectively, [38]. Additionally, between 2011 and 2012, the resistance to meropenem and imipenem was 7% and 9%, respectively, [39]. The increase in antibiotic resistance in these isolates was also well illustrated by the detection of β-lactamases. Isolates expressing different ESBLs were verified using a double-disk potentiation method, and three studies from the United Arab Emirates documented the phenotypic prevalence of ESBLs in *E. coli* and ESKAPE pathogens. These studies showed that during the study period (2000–2020), ESBL rates increased significantly from 36.2 to 75% [38,40,41].

The PCR analysis of various β-lactamase-encoding genes was carried out; the results shown in Table 2 indicate that *bla*_CTX-M_ was the most common ESBL enzyme among *E. coli* and ESKAPE pathogens, which was 21.3% in 2008 [41]. Other relatively less prevalent β-lactamase-genes were *bla*_OXA_, which was 6.4% in 2011/2012 and increased to 53.3% in 2015/2016 [39,42], presence of *bla*_SHV_ was also demonstrated during the study period. These studies showed that *bla*_NDM_ was the prevalent metallo-β-lactamase in the UAE.

### 3.3. Qatar

Seven single or multiple hospital-based surveillance studies were performed to assess the prevalence of antimicrobial resistance among *E. coli* and ESKAPE pathogens from Qatar, including 770 isolates of which *E. coli* isolates were the highest (*n* = 448) followed by *K. pneumoniae* (*n* = 168), *P. aeruginosa* (*n* = 106), *A. baumannii* (*n* = 48). Most of these isolates were recovered from patients admitted to the ICU and patients with cUTI or cystic fibrosis. During 2000–2020, antibiotic resistance was significantly increased in Qatar; the data presented in Table 1 show that resistance to 3rd- and 4th-generation cephalosporins; ceftazidime, and cefepime along with commonly used β-lactam/beta-lactamase inhibitor combinations, amoxicillin-clavulanic acid and piperacillin-tazobactam. Higher resistance was also seen for aminoglycosides; gentamycin and amikacin (aminoglycosides), ciprofloxacin (quinolone), while relatively lower resistance was recorded for meropenem (carbapenem) [43,44,45]. The increase in antibiotic resistance in these isolates was well substantiated by the identification of β-lactamases. The isolates expressing different ESBLs were verified using a double-disk potentiation method, while studies from Qatar documented the phenotypic prevalence of ESBLs in *E. coli* and ESKAPE pathogens. Those studies showed that during the study period, ESBL prevalence ranged between 9% to 31.7% based on the data with clinical isolates (*n* = 2166) [44,46,47]. Molecular characterization with the use of PCR demonstrated that *bla*_OXA_ and *bla*_CTX-M_ were the most prevalent ESBL traits among *E. coli* and ESKAPE pathogens, a smaller number of clinical isolates were reported to express *bla*_TEM_ and, *bla*_SHV_ ESBL traits (Table 2) [44,46,47,48].

### 3.4. Kuwait

Nine single or multiple hospital surveillance studies were performed to evaluate the prevalence of antimicrobial resistance among *E. coli* and ESKAPE pathogens, which included 2233 isolates of which *E. coli* isolates were the highest (*n* = 1206) followed by *K. pneumoniae* (*n* = 669), *P. aeruginosa* (*n* = 79), *A. baumannii* (*n* = 63), and *Enterobacter* spp. (*n* = 7). Most of these isolates were recovered from patients admitted to the ICU and blood samples. In Kuwait, during the period 2000 through 2020, increasing resistance to 3rd-generation cephalosporins (cefuroxime, and cefepime), and β-lactam/β-lactamase inhibitor (piperacillin-tazobactam), carbapenem (imipenem and meropenem) along with aminoglycosides (gentamicin and amikacin) and quinolones (ciprofloxacin) were becoming prevalent (see Table 1) [49,50,51]. These increasing trends of antibiotic resistance were also studied by the detection of β-lactamases in these isolates. Studies from Kuwait documented the phenotypic prevalence of ESBLs in *E. coli* and ESKAPE pathogens by the double potentiation method. These studies showed that the phenotypic prevalence of ESBL was between 31.7% and 32.8% in *E. coli* and ESKAPE pathogens [50,52]. In addition, the PCR amplification of various β-lactamases-resistance genes revealed that the most prevalent ESBLs distributed among *E. coli* and ESKAPE pathogens expressed the *bla*_CTX-M_ gene, which was 74% in 2009 [53]. Similarly, the prevalence of *bla*_OXA_ among *E. coli* and ESKAPE pathogens varied from 11.4 to 100% [50,54], for *bla*_VEB_ 2% [55] and for *bla*_NDM_ 34.4% [50].

### 3.5. Oman

Four single or multiple hospital-based surveillance studies were performed to assess the prevalence of antimicrobial resistance among *E. coli* and ESKAPE pathogens. These studies comprised 607 isolates, of which *E. coli* isolates were the highest (*n* = 165) followed by *K. pneumoniae* (*n* = 112), *P. aeruginosa* (*n* = 48), *S. aureus* (*n* = 155), *A. baumannii* (*n* = 107), and *Enterobacter* spp. (*n* = 20). Most of these isolates were recovered from patients admitted to the ICU. These studies reported a significant increase in resistance rates to various antimicrobial agents over the last 20 years. The results shown in Table 1 indicate that in Oman, higher resistance to piperacillin-tazobactam was common during 2000–2020, while relatively lower resistance was reported against cefuroxime, amoxycillin-clavulanate, and aminoglycosides, such as gentamicin and amikacin, and quinolones (ciprofloxacin) [56,57,58].

The increase in antibiotic resistance in these isolates was also explained by the detection of β-lactamase enzymatic activity and encoding genes. Isolates expressing various ESBL activity were verified among *E. coli* and ESKAPE pathogens using a double-disk potentiation method. Such research showed that during the study period (2000–2020), the phenotypic prevalence of ESBL was between 5% and 14.9% in *E. coli* and ESKAPE pathogens [57,58]. Additionally, molecular studies showed that between 2010 and 2011, the prevalence of *bla*_OXA_ and *bla*_NDM_ β-lactamase genes among *E. coli* and ESKAPE pathogens was 24.5% and 50%, respectively, [59].

### 3.6. Bahrain

Three single or multiple hospital-based surveillance studies were performed to assess the prevalence of antimicrobial resistance among *E. coli* and ESKAPE pathogens. These studies comprised 2420 isolates, of which *E. coli* isolates were the highest (*n* = 1594) followed by *K. pneumoniae* (*n* = 704), *P. aeruginosa* (*n* = 60), and *Enterobacter* spp. (*n* = 62). The majority of these isolates were recovered from patients admitted to the ICU. In the last 20 years, a substantial increase in the resistance level of different antimicrobial agents has been recorded. In 2019, resistance to ciprofloxacin was 100%, ceftazidime 86%, amikacin 72%, gentamicin 86%, meropenem 90%, imipenem 80% and piperacillin-tazobactam 90% [60]. The increase in antibiotic resistance in these isolates was also well demonstrated by the detection of β-lactamases enzymatic activities or genes.

As in other studies, isolates expressing ESBL activity were verified by the double-disk potentiation method. Studies from Bahrain revealed the phenotypic prevalence of ESBLs in *E. coli* and ESKAPE pathogens. Such research showed that the phenotypic prevalence of ESBLs during the study period (2000–2020) was 22.6% [61]. Furthermore, the PCR mediated amplification of various β-lactamases genes was also undertaken. In 2011, a study conducted showed that *bla*_SHV_, *bla*_TEM_ and *bla*_CTX-M_ β-lactamases encoding genes were the most predominant markers among *E. coli* and ESKAPE pathogens and accounted for 37%, 36.1% and 36.1%, respectively [62]. Another study conducted in Bahrain in 2011 showed that *bla*_VIM_ and *bla*_NDM_ were present in 47.5% and 2.5% of *E. coli* and ESKAPE pathogens [60] and *bla*_VIM_ was the most common metallo-β-lactamase encoding gene identified.

### 3.7. Yemen

Three single or multiple hospital-based surveillance studies were performed to determine the prevalence of antimicrobial resistance among *E. coli* and ESKAPE pathogens. These studies included 143 isolates, of which *E. coli* were the highest (*n* = 130) followed by *K. pneumoniae* (*n* = 8), *A. baumannii* (*n* = 3) and *Enterobacter* spp. (*n* = 2). These studies demonstrated slightly higher levels of resistance to various antimicrobials. For example, between 2014 and 2018, the resistance to cefuroxime was 95%, amoxycillin-clavulanate 90.9%, ciprofloxacin 90.9%, meropenem 95% and imipenem 95% [63,64]. The increase in antibiotic resistance in these isolates was also justified by the detection of β-lactamases. Isolates expressing various ESBL phenotypes were observed using a double-disk potentiation method. Studies from Yemen revealed the phenotypic prevalence of ESBLs in *E. coli* and ESKAPE pathogens. These studies showed that during the study period the phenotypic prevalence of ESBL increased from 33% to 100% [63,64]. Furthermore, analysis based on PCR studies was also undertaken. Bacteria expressing *bla*_SHV_, *bla*_TEM_ and *bla*_CTX-M_ were the most predominant ESBLs among *E. coli* and ESKAPE pathogens. In 2014, *bla*_SHV_ was found in 80% of the population, *bla*_TEM_ in 70%, *bla*_CTX-M_ in 80% [63] and *bla*_OXA_ in 10% [65]. This study showed that the prevalence of *bla*_NDM_ was 100% in 2014 [63].

### 3.8. Jordan

There were four single or multiple hospital-based surveillance studies, which evaluated the prevalence of antimicrobial resistance among *E. coli* and ESKAPE pathogens. Most of these isolates were recovered from ICU patients with cUTI and respiratory tract infections (RTI). Changing trends of antibiotic resistance in Jordan were also substantiated by data providing the phenotypic presence of β-lactamases. Isolates showing phenotypic expression of various ESBLs were verified using a double-disk potentiation method. These studies showed that in the study period, ESBL prevalence was relatively low (22.6% in 2009), but increased dramatically to 62% in 2019, as reported by a Jordan-based study on data obtained from 950 clinical samples [66,67]. Molecular biology studies demonstrated that *bla*_CTX-M_ was the most prevalent ESBL gene among *E. coli* and ESKAPE pathogens. It was detected in 25% of the population in 2000 and reached 68.9% in 2020 [67,68,69] while blaSHV increased from 12.5% to 20%. *bla*_TEM_ and *bla*_VEB_ both accounted for 18.9% of the population [67,68,69], and between 2012 and 2013, *bla*_OXA_ and *bla*_NDM_ were found in 71.5% and 28.5% of *E. coli* and ESKAPE bacteria, respectively, [69].

### 3.9. Iraq

Five single or multiple hospital-based surveillance studies were performed to assess the prevalence of antimicrobial resistance among *E. coli* and ESKAPE pathogens, which included 382 isolates of which *E. coli* isolates (*n* = 49), *K. pneumoniae* (*n* = 98), *P. aeruginosa* (*n* = 196), *S. aureus* (*n* = 17), *A. baumannii* (*n* = 9), and *Enterobacter* spp. (*n* = 13). The majority of these isolates were recovered from ICU hospitalized patients and patients with cUTIs. The results shown in Table 1 indicate that in Iraq during 2000 through 2020, resistance to 3rd- and 4th-generation standalone cephalosprins; ceftazidime, and cefepime was higher than that of β-lactam/β-lactamase inhibitor combination, amoxicillin-clavulanic acid. Curiously, resistance to piperacillin-tazobactam combination, aminoglycosides (gentamicin and amikacin) was higher than carbapenems such as; imipenem and meropenem [70,71,72,73]. These increases in antibiotic resistance in these isolates were correlated with the identification of β-lactamases phenotypically. Isolates expressing various ESBLs were verified by using a double-disk potentiation method. Studies based on the 402 clinical samples have revealed that the incidence of ESBLs pathogens was comparatively low (13.5%) in 2000, but significantly increased to 61.5% over time [70,72,73]. The results shown in Table 2 represent molecular characterization of the ESBL trait with PCR analysis. Table 2 shows that *bla*_SHV_, *bla*_TEM_ and *bla*_CTX-M_ were the most predominant ESBLs among *E. coli* and ESKAPE pathogens, and an increasing trend was also observed for *bla*_OXA_, *bla*_PER_, and *bla*_VEB_ ESBL traits. *bla*_NDM_ was the most prevalent metallo-β-lactamase encoding gene, with fewer isolates demonstrating expression of the *bla*_VIM_ and *bla*_IMP_ ESBL trait [71,72,73,74].

### 3.10. Syria

Extremely limited antibiotic surveillance data are available for Syria. They are limited to two single or multiple hospital-based surveillance studies comprising of 364 isolates of which 104 were *E. coli* and 260 *A. baumannii*. The majority of these isolates were recovered from ICU hospitalized patients and patients with UTIs. One study showed a significant increase in resistance to various antimicrobial agents over the last 20 years. Between 2008 and 2011 resistance to cefotaxime reached 91.4%, ceftazidime 80.6%, cefepime 84.7%, amoxycillin-clavulanate 93.2%, ciprofloxacin 81.2%, amikacin 78.3%, gentamicin 82.7%, piperacillin-tazobactam 91.2%, and aztreonam 98.5% [75]. Moreover, isolates expressing various ESBLs were verified using a double-disk potentiation method. Studies from Syria documented the phenotypic prevalence of ESBLs among *E. coli* and ESKAPE pathogens. These studies showed that the phenotypic prevalence of ESBL was 52% [76].

### 3.11. Lebanon

Five single or multiple hospital monitoring studies were conducted to assess the prevalence of antimicrobial resistance among *E. coli* and ESKAPE pathogens including 2837 isolates of which *E. coli* isolates were highest (*n* = 2000) followed by *K. pneumoniae* (*n* = 676), *P. aeruginosa* (*n* = 40) and *A. baumannii* (*n* = 121). Most of these isolates were recovered from ICU hospitalized patients and patients suffering from cUTIs. The results shown in Table 1 indicate that in Lebanon, during 2020 through 2020, resistance to 3rd- and 4th-generation cephalosporins, such as cefotaxime, ceftazidime, and cefepime, aminoglycosides, e.g., gentamicin and amikacin, ciprofloxacin—a quinolone drug—and carbapenems, such as imipenem and meropenem, was higher. Curiously, resistance to piperacillin-tazobactam was relatively lower [77,78,79,80]. The increasing antibiotic resistance trends in Lebanon were justified by characterization of β-lactamase phenotypic and genotypic activities. Isolates expressing various ESBLs were tested using a dual-disk potentiation method. ESBL prevalence was relatively low (24%) in 2000 but increased dramatically to 84% in 2020 [78,81]. Molecular analysis carried with PCR, shown in Table 2 indicated that *bla*_SHV_, *bla*_TEM_ and *bla*_CTX-M_ were the most predominant ESBL-encoding genes among *E. coli* and ESKAPE pathogens, this increasing trend was also observed for *bla*_OXA_, and *bla*_GES_ ESBL traits. On the other hand, *bla*_VIM_ was the most common metallo-β-lactamass resistance encoding gene which showed remarkable increase, with fewer isolates shown to express the *bla*_NDM_ ESBL trait [77,78,79,80,81].

### 3.12. Palestine

Limited antibiotic susceptibility data are available from only two single hospital-based surveillance studies. Antimicrobial resistance among *E. coli* and ESKAPE pathogens comprised 119 isolates, out of which 101 were *E. coli* followed by *K. pneumoniae* (*n* = 15), and *Enterobacter* spp. (*n* = 3). The majority of these isolates were recovered from ICU hospitalized patients. These studies reported significantly increased resistance rates to various antimicrobial agents during the years 2000–2020. The results shown in Table 1 indicate that in Palestine during 2000 to 2020, resistance to 3rd-generation cephalosporins, i.e., cefuroxime, cefotaxime, ceftazidime, was higher. Interestingly, lower resistance was reported against amoxicillin-clavulanic acid, aminoglycosides (gentamicin and amikacin), quinolones (ciprofloxacin), and imipenem [82,83]. Isolates expressing various ESBLs, characterized by double-disk potentiation method, showed relatively low ESBL rates 20 years ago (35.3%), which increased dramatically to 85.3% [82,83]. The molecular studies shown in Table 2 indicate that *bla*_CTX-M_ was the most predominant ESBL trait among *E. coli* and ESKAPE pathogens, which evolved from 71.4% to 100%. A similar upward trend was also observed for *bla*_TEM_, *bla*_OXA_ and *bla*_SHV_ ESBL traits [82,83].

### 3.13. Egypt

Six single or multiple hospital-based surveillance studies were carried out to evaluate the prevalence of antimicrobial resistance among *E. coli* and ESKAPE pathogens. These studies comprised 1289 isolates, of which *E. coli* were the highest (*n* = 661), followed by *K. pneumoniae* (*n* = 246), *P. aeruginosa* (*n* = 356), *A. baumannii (n* = 7), and *Enterobacter* spp. (*n* = 19). Most of these isolates were recovered from ICU inpatients and patients with complex urinary tract infections. The results shown in Table 1 indicate that in Egypt from 2000 through 2020, resistance to various cephalosporins, such as cefuroxime, cefotaxime, ceftazidime, and cefepime, was higher than that to b-lactam/b-lactamase inhibitor combinations, e.g., amoxicillin-clavulanic acid and piperacillin-tazobactam. A rise in resistance was reported against aminoglycosides; gentamcin/amikacin, ciprofloxacin (a quinolone drug) and carbapenems, i.e., imipenem and meropenem [84,85,86,87,88,89]. The increase in antibiotic resistance in these isolates has also been demonstrated by β-lactamase detection. These studies from Egypt reported the phenotypic prevalence of ESBLs in *E. coli* and ESKAPE pathogens with ESBL rate of 7.4%, which increased to 48.9% [84,89,90,91,92]. Molecular characterization of the ESBL trait with PCR showed that *bla*_SHV_, *bla*_TEM_ and *bla*_CTX-M_ were the most predominant traits among *E. coli* and ESKAPE pathogens. An increasing trend of *bla*_OXA_ ESBL was also documented during 2000–2020, as shown in Table 2. An increasing trend was also observed for *bla*_PER_ and *bla*_GES_ ESBL traits. On the other hand, *bla*_VIM_ and *bla*_IMP_ were the most prevalent metallo-β-lactamases resistance encoding genes which showed a remarkable increase, along with *bla*_NDM_ [84,88,90,91,92,93].

### 3.14. Libya

Based on susceptibility studies undertaken by two single or multiple tertiary care hospitals from Libya, the prevalence of antimicrobial resistance among *E. coli* and ESKAPE pathogens was determined. These studies involved 433 isolates, of which *E. coli* were the highest (*n* = 397) followed by *K. pneumoniae* (*n* = 36). Most of these isolates were recovered from ICU inpatients. These studies reported significantly increased resistance rates to β-lactams. For instance, resistance to cefotaxime was 93%, ceftazidime 93% and cefepime was 93% [87]. Isolates expressing various ESBLs were confirmed using a dual-disk potentiation method, wherein ESBL prevalence was found to be 66.6–67.6% [87,94]. Furthermore, analysis based on molecular studies involving PCR mediated amplification of various β-lactamases showed that *bla*_CTX-M_ (51.7 to 85.9%) were the most predominant ESBLs in *E. coli* and ESKAPE pathogens [87,94], with *bla*_SHV_ at 21.8%, *bla*_TEM_ 35% and *bla*_OXA_ 11.4% in 2016 [94].

### 3.15. Algeria

With four single or multiple hospital-based surveillance studies, the prevalence of antimicrobial resistance among *E. coli* and ESKAPE pathogens was assessed. Overall, 668 isolates were found in these studies, of which *E. coli* (*n* = 232) and *K. pneumoniae* (*n* = 246) were the highest, along with *S. aureus* (*n* = 106), *A. baumannii* (*n* = 17), and *Enterobacter* spp. (*n* = 67). Most of these isolates were recovered from ICU inpatients and patients with complex urinary tract infections. These studies reported significantly increased resistance rates to various β-lactam antibiotics. For instance, in 2008 cefuroxime resistance was 87.3%, cefotaxime 59%, aztreonam 33.3%, and ceftazidime 12.8% [95]. Isolates expressing various ESBLs were tested using a dual-disk potentiation method, which showed increased ESBL rates from 17.7% to 47.6% during the study period [95,96,97]. Additionally, based on the molecular analysis, it was observed that in 2009, *bla*_SHV_ was 10% [96], *bla*_TEM_ 19.9% to 70% [95,96,98], *bla*_CTX-M_ 19.9% to 100% [95,96,97,98], and in 2013 *bla*_VIM_ was 82.3% [99].

### 3.16. Tunisia

A total of 1650 *E. coli* and ESKAPE pathogens, *E. coli* (*n* = 368), *K. pneumoniae* (*n* = 811), *P. aeruginosa* (*n* = 210), *A. baumannii* (*n* = 246), and *Enterobacter* spp. (*n* = 15), were recovered from six single or multiple hospital-based surveillance studies to assess the prevalence of antimicrobial resistance in these pathogens. Most of these isolates were recovered from ICU inpatients. The results shown in Table 1 indicate that in Tunisia during 2000 to 2020 resistance to 3rd-generation cephalosporins, such as cefuroxime and ceftazidime, aminoglycosides, e.g., gentamicin and amikacin, was higher than resistance to ciprofloxacin, a quinolone drug. Curiously, most reports showed susceptibility to the carbapenem drug imipenem [100,101,102,103]. Based on phenotypic methods, the prevalence of ESBLs in *E. coli* and ESKAPE pathogens was determined, which was 7.3% in year 2000 and reached 100% [101,102,103,104]. Molecular data showed that *bla*_SHV_, *bla*_CTX-M_ and *bla*_TEM_ were the most predominant ESBLs in *E. coli* and ESKAPE pathogens, which were 57.3% and increased to 92.1% [100,101,102], from 6.6% to 83.3% [100,101,102,103] and 0.6 to 51.6% [100,101,102,103], respectively. A similar increase in β-lactamase was observed for *bla*_OXA_—50% to 93.9% [100,101,105], and between 2013 and 2015, *bla*_NDM_ was 4.4% [105].

### 3.17. Morocco

Antibiotic resistance data were available for 904 clinical isolates, of which *E. coli* was the highest (*n* = 639) along with some *K. pneumoniae* (*n* = 113), *P. aeruginosa* (*n* = 123), and *Enterobacter* spp. (*n* = 15). These data were based on four single or multiple hospital-based surveillance studies and the majority of isolates were from ICU patients. Between 2012 and 2014, studies showed resistance to ceftazidime 8.8%, ciprofloxacin 39.5%, amikacin 11.8%, gentamicin 48.5%, imipenem 36.8%, meropenem 27.9%, piperacillin-tazobactam 9.8%, and aztreonam 35.3% [106]. The increase in antibiotic resistance in these isolates was also well demonstrated by β-lactamase detection. Isolates expressing various ESBLs were tested using a dual-disk potentiation method. Moroccan studies reported the phenotypic prevalence of ESBLs in *E. coli* and ESKAPE pathogens. These studies showed that, in the study period, ESBL prevalence was 1.3%, which increased to 58% [107,108,109]. In addition, molecular studies showed that *bla*_SHV_, *bla*_CTX-M_, *bla*_TEM_ were the most predominant ESBLs in *E. coli* and ESKAPE pathogens, which showed a dramatic rise during the study period such as 9.1% in 2000 increasing to 67.3% in 2020 [108,109], *bla*_TEM_ 42.8% [108], *bla*_CTX-M_ 1.1 to 90.9% [107,108,109], *bla*_OXA_ 13% [109], and *bla*_VIM_ 6% [106].

### 3.18. Sudan

To assess the prevalence of antimicrobial resistance among *E. coli* and ESKAPE pathogens, three single or multiple hospital-based surveillance studies were conducted. These studies contain 823 isolates, of which *E. coli* (*n* = 450), *K. pneumoniae* (*n* = 315), *S. aureus* (*n* = 54), and *A. baumannii* (*n* = 4). Most of these isolates were obtained from ICU patients. These studies reported increased resistance rates to various antimicrobial agents. For instance, resistance to ceftazidime 55.6% [110], ciprofloxacin 81.4% [111], amikacin 95.7% [111], cefotaxime 61.1% to 89% [110,111]. The increase in antibiotic resistance in these isolates has also been well demonstrated by β-lactamase detection. Isolates expressing various ESBLs were tested using a dual-disk potentiation method. Studies from Sudan reported the phenotypic prevalence of ESBLs among *E. coli* and ESKAPE pathogens. These studies showed that in the study period, ESBL prevalence ranged from 30.2% to 44.4% [110,111]. In addition, research was also conducted based on molecular experiments involving PCR mediated amplification of various β-lactamases. These studies showed that *bla*_VIM_ was the most predominant MBLs in *E. coli* and ESKAPE pathogens, which were *bla*_VIM_ 38.9%, *bla*_IMP_ 36.4%, and *bla*_NDM_ 4.2% during 2015 to 2016 [112].

### 3.19. Djibouti

One single hospital-based surveillance study was conducted to assess the prevalence of antimicrobial resistance among *E. coli*. Limited data is available, with only 31 *E. coli* isolates. Based on the molecular data in 2019 involving PCR mediated amplification showed *bla*_TEM_ 10% and *bla*_CTX-M_ 96.8% [113].

## 4. Discussion

**Figure 2:**
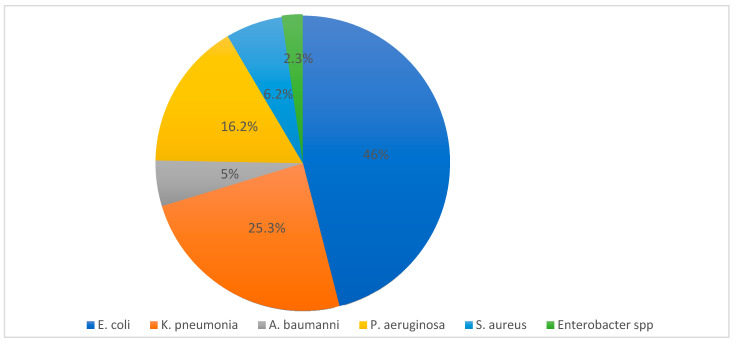
Percentage of distribution of *E. coli* and ESKAPE pathogens in the Arab region during 2000–2020.

As shown in Table 3 and Figure 2, 19 of 22 Arab countries (86.3%) provided antimicrobial resistance data on *E. coli* and ESKAPE pathogens, especially Gram-negatives. Similar data was not found for three countries: Mauritania, Somalia, and the Comoros Islands. Between January 2000 and February 2020, there were 109 articles published reviewing antibiotic resistance in Arab region countries. Among the *E. coli* and ESKAPE pathogens, the predominantly encountered pathogen was *E. coli* (*n* = 32,038), followed by *K. pneumoniae* (*n* = 17,128), *P. aeruginosa* (*n* = 11,074), methicillin-resistant *S. aureus* (MRSA, *n* = 4370), *A. baumannii* (*n* = 3485) and *Enterobacter* spp. (*n* = 1574). Overall, the most prevalent Gram-negative pathogens in Arab regions were *E. coli* and *Klebsiella* spp., mainly encountered in respiratory infections, urinary tract infections and blood stream infections. *P. aeruginosa* and *A. baumannii* were mainly from respiratory infections associated with hospital acquired bacterial pneumonia and ventilator associated bacterial pneumonia in ICU settings. Meanwhile, infections caused by *Enterobacter* spp. were mainly reported from complicated digestive tract and urinary tract infection. To understand the antibiotic resistance trends in Gram-negative isolates, data containing a higher proportion of isolates from Saudi Arabia, Kuwait, Oman, Bahrain, Qatar, UAE, Egypt Sudan and Tunisia could be more helpful.

**Figure 3:**
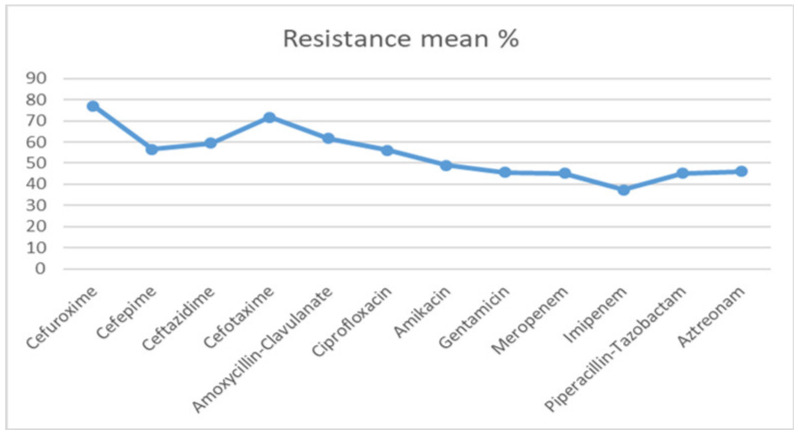
Antibiotic resistance among *E. coli* and ESKAPE pathogens in the Arab region (mean percentage) during 2000–2020.

The data from these countries showed the distribution of *E. coli* and ESKAPE pathogens as shown in Figure 3. *E. coli* was highest, at 46%, followed by *K. pneumoniae*, 25.3%, and then *P. aeruginosa*, 16.2%, *S. aureus*, 6.2%, *A. baumannii*, 5%, and *Enterobacter* spp., 2.3%.

Additionally, the data from these countries showed significant increased resistance to cephalosporins such as cefuroxime, cefotaxime, ceftazidime, cefepime and a β-lactam and β-lactamase inhibitor combinations such as amoxicillin-clavulanic acid and piperacillin-tazobactam in the last 20 years. The mean values of resistance rates to cephalosporins (cefuroxime, cefotaxime, ceftazidime, and cefepime), and β-lactam and β-lactamase inhibitor combinations (amoxicillin-clavulanic acid and piperacillin-tazobactam) among *E. coli* and ESKAPE pathogens were (77.1%, 71.8%, 59.5%, and 56.5%), and (61.7% and 45.3%), respectively. Similarly, higher resistance rates were observed for aminoglycosides (gentamicin and amikacin), quinolones (ciprofloxacin), and carbapenems (imipenem and meropenem) among *E. coli* and ESKAPE pathogens was (45.7% and 48.9%), (56.2%), and (37.5% and 45.2%), respectively. As shown in Figure 3, in *E. coli* and ESKAPE pathogens, resistance to imipenem and meropenem did not vary much during the study period and were lowest among all.

Overall, the antibiotic resistance rates in Arab regions are significantly higher than those observed in the USA and Europe. Based on recently published global surveillance studies, SENTRY involving a significantly high proportion of *E. coli, K. pneumoniae, P. aeruginosa, S. aureus, Enterobacter* and *A. baumannii* were collected during the years 2013 through 2018 by JMI labs. We compared resistance trends in these nations against Arab nations. Our analyses of antibiotic resistance rates in Arab regions during the study period (2000 to 2020) showed mean values of resistance rates in *E. coli* to cephalosporins (cefuroxime, ceftazidime, and cefepime) as (85.1%, 63.8%, and 66.3%), respectively, while in the USA they were about 38.5%, 11%, and 11.7%, respectively, and in the UK 35.9%, 10%, and 11.1%, respectively. In other countries such as Australia, they were 32%, 9.1%, and 9.8%, respectively. Considering the resistance to β-lactam and β-lactamase inhibitor combinations (amoxicillin-clavulanic acid and piperacillin-tazobactam), it was (58.8% and 40.1%), respectively, in Arab nations. However, significantly lower resistance was observed in the US (21.6% and 4%) and UK (27.6%, and 6.8%). In developing nations such as China (35% and 7.9%) and India (59.6% and 21.3%), these values were relatively higher. Similarly, resistance rates to aminoglycosides such as gentamicin and amikacin were 52.8% and 43.1%, respectively, in Arabic countries, while in the US they were 12.4% and 0.3% and in the UK they were 10.8 and 0.8%, which is significantly lower. In Australia, they were (8.4% and 0.2%), China (54.5% and 3.4%) and India (41.3% and 6.4%), respectively, while in the US, UK, China and India they reached 32.4%, 24.3%, 72% and 72.3%, respectively. Resistance to carbapenems (imipenem and meropenem) in *E. coli* was 33.6% and 47.8%, respectively, while in the US and UK it was 0.2% for both. In Australia, it was remarkably low (0.1% for imipenem and 0% for meropenem). In China, it was about 1.1% and 0.1%, respectively, and in India, 8.5% and 8.5%, respectively, [114]. Similar resistance trends were noted in *K. pneumoniae* in other territories such as the US, and Europe, which were significantly lower than Arabic nations. In *K. pneumoniae*, cephalosporin resistance in the US and Europe was in the range of 12–31%. However, in Arabic regions, this was significantly higher, at 63–86%. Resistance to aminoglycosides such as amikacin and gentamicin (42–64%) and carbapenems (27–36%) was relatively higher in Arabic nations, while in the US and Europe, it was about 2–8% and 1–4%, respectively. It is important to note that the resistance rates to aminoglycosides and carbapenems in Arabic nations are higher than in India (24–34% and 29–30%) and China (11–29% and 12–13%) (SENTRY 2013–2018) [114]. Additionally, the mean values of resistance rates in *P. aeruginosa* to cephalosporins (ceftazidime and cefepime) were 62% and 58.2%, respectively, in Arabic nations, while in the US they were 15.8% and 15.2%, respectively, and in the UK they were 10.7% and 8.2%, respectively. In Australia they were only 12.6% and 8.3%, respectively, and resistance to β-lactam and β-lactamase inhibitor combinations such as piperacillin-tazobactam was 51.2% in Arabic countries, while it was 19.7% in the US, 13.7% in the UK, 15.5% in Australia, 31.3% in China and 40.5% in India. Similarly, the resistance rates to aminoglycosides (gentamicin and amikacin) were 62.6% and 51.4% in Arab nations, while in the US they were 14% and 4%, and in the UK 3.7% and 1%, respectively. In Australia, they were only 8.1% and 4.6%, in China 15.2% and 7.4%, and in India 40.5% and 27.9%, respectively. The resistance to quinolones (ciprofloxacin) was 58.3% in Arab nations. In the US, it was 28.2%, UK 12.2%, Australia 14.8%, China 29.6% and India 43.3%. The resistance to carbapenems (imipenem and meropenem) in *P. aeruginosa* was 42.6% and 35.6%, respectively, while in the US and the UK it was much lower (21.8% and 19.8%, and 17% and 15.2%, respectively). In Australia, it was 12% and 8.6%, respectively, China 33.3% and 27.7%, respectively, and India 33.4% and 33.4%, respectively [114]. Therefore, the overall antibiotic resistance to *P. aeruginosa* is quite similar across various geographies owing to the limited therapeutic options available for the treatment of infections caused by these pathogens. Based on the availability of antibiotic susceptibility, combinations of piperacillin-tazobactam or imipenem/meropenem and aminoglycosides are administered for the treatment of these difficult-to-treat pathogens. However, the increasing resistance to these higher antibiotics is a serious threat in hospital settings. Likewise, the mean values of resistance rates in *S. aureus* to cephalosporins, cefepime was 50% in Arab countries, while in the USA it was 48%, 18.5% in UK, and 31.4% in Australia. Resistance to β-lactam and β-lactamase inhibitor combinations (amoxicillin-clavulanic acid and piperacillin-tazobactam) affected 50% and 54.4% of isolates, respectively, while in the USA it was 48% and 46.2%, respectively, UK 18.8% and 16.3%, Australia 31.3% and 28.1%, China 45.7% and 45.7% and India 51.3% and 51.3%. Similarly, the resistance to quinolones (ciprofloxacin) was 38.3%, while in the USA, UK, Australia and China; it was 40.6%, 19.4%, 17.4% and 46.9%, respectively. The phenotypic resistance to carbapenems (imipenem and meropenem) in *S. aureus* were 22.5% and 25.6%, respectively, while in the US it was 48% and 48%, respectively, and in the UK 18.4% and 18.5%, respectively, Australia 31.4% and 31.4%, respectively, China 45.7% and 45.7%, respectively, and India 51.3% and 51.3%, respectively (SENTRY 2013–2018) [114]. *A. baumannii* is another highly challenging pathogen owing to its ability to intrinsically express diverse resistance mechanisms such as highly reduced outer membrane porins and the ability to express the efflux pumps in higher order, leading to insufficient concentration of antibiotics inhibiting the bacterial cell targets. Therefore, antibiotic resistance to *A. baumannii* is significantly higher around the world. The mean values of resistance rates in *A. baumannii* to cephalosporins (ceftazidime and cefepime) were 91.3% and 79%, respectively, while in the USA 45.3% and 49.9%, respectively, and UK 7.2% and 21.4%, respectively, Australia 18.8% and 27.5%, respectively, and β-lactam and β-lactamase inhibitor combinations such as piperacillin-tazobactam were 60%, while in the USA, UK, Australia, China and India, they were 52.2%, 28.6%, 28.1%, 82.4% and 55.6%, respectively. Similarly, resistance rates of aminoglycosides (gentamicin and amikacin) were 73.3% and 72.2%, while in the US they were 37.9% and 23.7%, respectively, and in the UK 35.7% and 14.3%, respectively, Australia 13% and 8.7%, respectively, China 79.5% and 72.9%, respectively, and India 50% and 55.6%, respectively. Additionally, resistance to quinolones (ciprofloxacin) was 86.4%, while in the US 48.6% and UK 14.3%, Australia 17.4%, China 79% and India 45.6%. The resistance to carbapenems (imipenem and meropenem) in *A. baumannii* was 60.7% and 62.3%, respectively, while in the US it was 39.9% and 42.2%, respectively, UK 14.3% and 14.3%, respectively, Australia 8.7% and 8.7%, respectively, China 75.9% and 77.7%, respectively, and India 55.6% and 55.6%, respectively [114]. Against another clinically important Gram-negative pathogen, *Enterobacter* spp., resistance to cephalosporins (ceftazidime and cefepime) was 66.8% and 4.4%, respectively, in Arabic regions, while in the US it was 23% and 8.4%, respectively, and UK 15.2% and 5.9%, respectively, Australia 26.2% and 8%, respectively. Resistance to β-lactam and β-lactamase inhibitor combination (piperacillin-tazobactam) was 48% in the Arab region, while in the US it was 17.8%, UK 8.8%, Australia 23.3%, China 23.3% and India 0%. Similarly, resistance rates to aminoglycosides (gentamicin and amikacin) in the Arab region were 62.8% and 41.4%, while in the US they were 4.4% and 0.3%, respectively, UK 3.5% and 0%, respectively, Australia 6% and 0%, respectively, China 16.4% and 1.4%, respectively, and India 33.3% and 0%, respectively. Additionally, resistance to quinolones (ciprofloxacin) in the Arab region was 50.4%, while in the US it was 9.3%, UK 2.3%, Australia 6%, China 26%, and India 33.3%. Resistance to carbapenems (imipenem and meropenem) among *Enterobacter* spp. isolates was 29.4% and 30.6%, respectively, while in the US it was 2.4% and 1.8%, respectively, in the UK 2.4% and 0.6%, respectively, Australia 2.5% and 1.5%, respectively, China 1.4% and 1.4%, respectively, and India 0% and 0%, respectively [114].

Table 4 provides antibiotic resistance trends at 5-year intervals. It shows a sustained increase in antibiotic resistance rates during the study period (2000–2020). It was low in the first period (2000–2005) and increased in the second period (2006–2010); this increase continued from 2011–2015 and reached the highest peak in the last period 2016–2020. For example, the mean values of resistance rates to cephalosporins (cefuroxime, cefotaxime, ceftazidime and cefepime were 44.6%, 62.5%, 49% and 39% in 2000–2005, which increased to 87.2%, 78%, 53% and 47% in 2006–2010. This increase continued to 89.2%, 74.3%, 62.8% and 66.5% in 2011–2015 and reached a maximum in the last period of 87.5%, 72.5%, 72.3% and 73.6% in 2016–2020, respectively.

**Figure 4:**
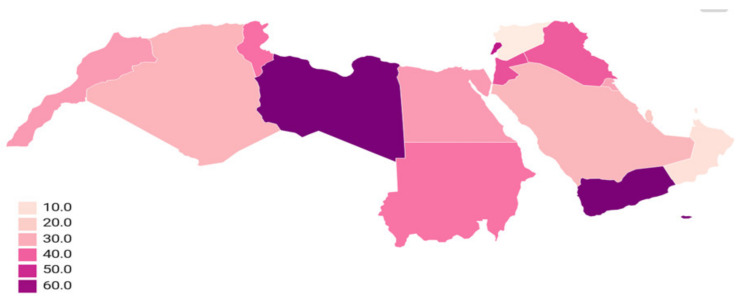
Geography distribution of ESBL among *E. coli* and ESKAPE pathogens in the Arab region (mean percentage) during 2000–2020.

Figure 4 shows the geographic distribution of ESBL among *E. coli* and ESKAPE pathogens in the Arab region (mean percentage). An average of 36.9% of total isolates were reported to have ESBL phenotypes. However, according to SENTRY antibiotic susceptibility surveillance studies (2013–2018) by JMI labs, the ESBL *E. coli* rates were very low, and those in the USA, UK and Australia were 15.2%, 15.2% and 14.5%, respectively. On the other hand, it was very high in China and India, at 65.8% and 68%, respectively [114].

**Figure 5:**
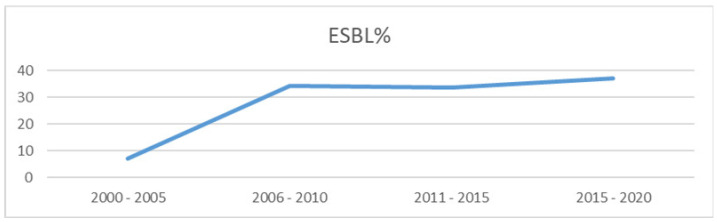
Comparing ESBL producing *E. coli* and ESKAPE Pathogens during 2000–2020.

Figure 5 provides ESBL rates every 5 years, showing a sustained increase in ESBL rates during the study period (2000–2020), it was (mean) 6.9% in 2000–2005, 34.4% in 2006–2010, 33.5% in 2011–2015 and 37.1% in 2016–2020.

**Figure 6:**
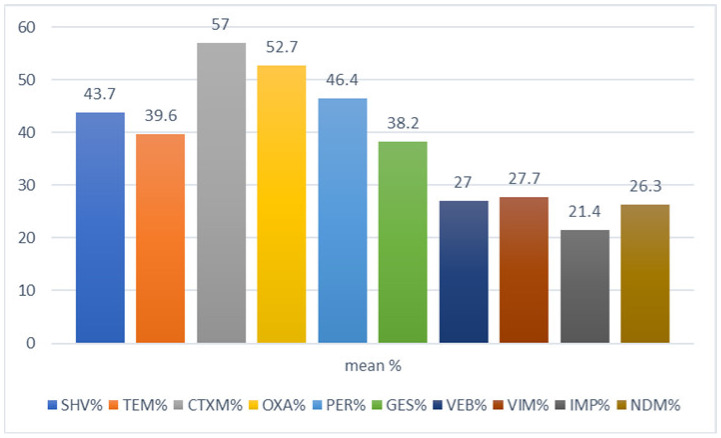
Distribution of β-lactamase genes among *E. coli* and ESKAPE pathogens in the Arab region.

The molecular data from the Arab region are abundant enough to suggest that the prevalence of antibiotic resistance among *E. coli* and ESKAPE pathogens is rising steadily with a predominance of *bla*_CTX-M_ and *bla*_OXA_ of ESBLs (Class A and D) and also *bla*_VIM_ and *bla*_NDM_ of MBLs (Class B) in most countries in the Arab region (Figure 6).

**Figure 7:**
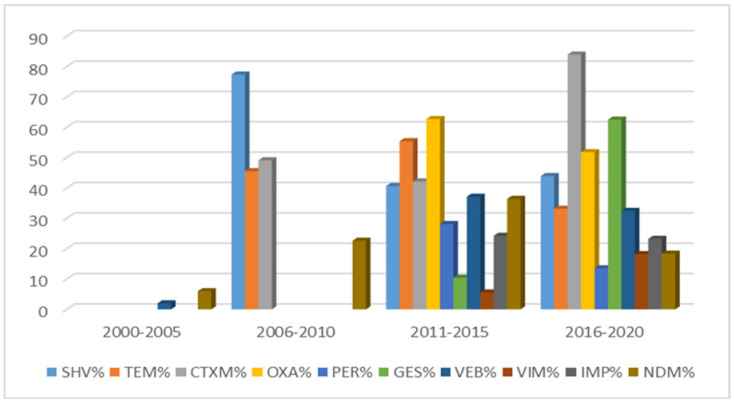
Comparing β-lactamase genes among *E. coli* and ESKAPE pathogens in the Arab region during 2000–2020.

This research indicates the predominance of *bla*_CTX-M_ and correlates this to *bla*_CTX-M_ enzymes that have spread globally [115]. Most studies from Arab region countries reported the molecular mechanisms of resistance among *E. coli* and ESKAPE pathogens. The most commonly produced β-lactamases were (mean) *bla*_CTX-M_ 57%, *bla*_OXA_ 52.7%, *bla*_PER_ 46.4%, *bla*_SHV_ 43.7%, *bla*_TEM_ 39.6%, *bla*_GES_ 38.2%, *bla*_VEB_ 27%, *bla*_VIM_ 27.7%, *bla*_IMP_ 21.4% and *bla*_NDM_ 26.3%. The molecular mechanism of β-lactamase showed levels sustaining a growing trend through the period of study (2000–2020), as shown in Figure 7.

These results are consistent with INFORM surveillance studies IHMA that reported CTX-M was the dominant ESBL in Europe with the exception of Greece, where SHV-type ESBLs were more common, with OXA-48 like, NDM and VIM-positive among *K. pneumoniae* from the European Union countries [116]. High inter-country differences in resistance rates between Arab nations are consistent with those in the European Union, according to the European Antimicrobial Resistance Surveillance Network (EARS-Net) report published in 2016. This report shows large differences in antimicrobial resistance patterns for different countries within the Arab region. Antibiotic resistance in southern and eastern European countries is higher than in north European ones [117]. Curiously, the occurrence of resistant isolates in the Arab region is higher than in other regions in Asia [118]. Overall, all of the Arabic countries showed relatively higher antibiotic resistance expressed in *E. coli* and Gram-negative ESKAPE pathogens compared to other developing countries such as India and China (SENTRY 2013–2018) [114]. Manifestation of such higher resistance could be due to the lack of access to standard healthcare, exposure to warlike scenarios, hot and dusty climatic factors, unsanitary living conditions and population factors, the status of disease resistance, and also the high prevalence of self-medication with antibiotics. Indeed, Rimah’s 2019 Gulf Cooperation Council Countries (GCCC) study revealed that self-medication of antibiotics ranged from 14% to 73%. Saudi Arabia posted the highest prevalence of self-medication (55%), followed by Kuwait (28%), Oman (18%) and Qatar (14%).

Based on adherence to antibiotic courses, it was found that a vast majority of patients, 30% to 72%, who were prescribed antibiotics did not comply [119]. In 2018, Maysun analyzed the attitude and actions of Palestinian refugees attending UNRWA health centers in Jordan concerning antibiotic use, finding that unreasonable antibiotic usage was widespread in 63% of patients who shared antibiotics at home, and 60% who bought antibiotics directly from the pharmacy without a prescription [120]. Syria’s research showed that 57% of patients used old prescriptions or took advice from someone else [121]. In Yemen, the prevalence of self-medication with antibiotics was 79.1% [122]. The indiscriminate use of these antibiotics drives selection pressure, enabling the emergence of more and more resistant strains. A word of caution is needed with respect to the use of antibiotics, as the majority of the resistance expressed and spread across various species is due to plasmid-mediated transmission of resistance genes. This study affirms the need for serious measures that are necessary to prevent antibiotic abuse, which would ultimately lead to infection control as well as selection and dissemination of antibiotic resistance. While the data provided in this review on antimicrobial resistance cover the commonly resistant Gram-negative pathogens in the Arab region, they are not dependent on formal national surveillance studies and are collections from the literature published by independent Arab region scientists. Consequently, *Enterococcus* spp. data were not included due to the limited data available.

Developing countries in the Arab region have poor medical and healthcare facilities, and this could be another potential reason for the prevalence and further spread of antibiotic resistance among *E. coli* and ESKAPE pathogens. Many developed countries, such as the United States, Europe, Australia, China and India, have their own national surveillance programs, which provide systematic antibiotic susceptibility information. This surveillance data helps clinicians to rationalize the deployment of empiric treatment in a more appropriate way. However, in the absence of such data, irrational use of antibiotics is promoted in developing countries like Arabic regions. Therefore, this study also highlights the need for national surveillance in individual countries in the Arab region, and this strategy should be a priority for national strategic health plans as part of a global antimicrobial resistance containment objective to obtain a clearer and more accurate picture of this situation. These studies were restricted to *E. coli* and ESKAPE pathogens, rather than Gram-negative bacteria in general, and this could be a limitation; however, these pathogens have been associated with frequent outbreaks and health care associated means of antibiotic resistance. Studies on the emergence and spread of antibiotic resistance from the developed world and developing countries has become essential. Further studies are required to dissect and reason why there is negligible occurrence of *Enterococcus faecium* in comparison to that in the developed world.

## 5. Conclusions

Increasing incidence of antibiotic resistance has been reported in various countries around the world, particularly in the Arab region. The recent emergence and increase of ESBL strains among *E. coli* and ESKAPE pathogens in this region is reaching an alarming level. The prevalence of ESBL bacteria is variable throughout the Arab region countries. A high prevalence of ESBL has been reported from most of the Arab region countries. It is particularly high in Libya, with an average of 67.1%, followed by Yemen, with an average of 66.9%, whereas it remains relatively lower in other Arab countries. The resistance appears to be commonly mediated by *bla*_CTX-M_ in these regions. Additionally, resistance mediated by *bla*_NDM_ was also observed. Additionally, socio-cultural health determinants associated with the structure and conditions of health care systems, as well as health seeking behaviors, are the main factors that influence self-medication with antibiotics. This study illustrates the need for national surveillance in individual countries in the Arab region, and this policy should be a priority for national strategic health plans as a part of the global antimicrobial resistance reduction target to get a better and more accurate picture of this situation

## Figures and Tables

**Table 1:** Increased resistance rates to antimicrobial agents in *E. coli* and ESKAPE pathogens in the Arabian region for the years 2000–2020.

Country	Increased Antimicrobial Resistance Rates during 2000–2020											
	CXM	GAZ	CPM	CTX	AUG	TPZ	CIP	AK	CN	MEM	IMP	ATM
Saudi Arabia	54% to 80%	16.6% to 95%	8.3% to 83%	4.4% to 97%	25% to 86%	4.9% to 33.3%	24% to 94.5%	23.5% to 82%	29% to 91.6%	0% to 57.4%	0% to 63.6%	4.9% to 99%
Qatar	-	93.6%	99%	-	92%	22% to 50%	34.4% to 91.7%	3% to 99%	28% to 99%	0% to 58.3%	-	-
Kuwait	100%	-	32.8%	-	-	32.8% to 97.8%	31.1% to 100%	23% to 93.5%	45%	54%	32.8% to 100%	-
Oman	-	-	-	24% to 45%	66%	22% to 99%	17.7% to 25%	12.7%	12.7%	-	-	-
Iraq		36.3% to 87%	50% to 78%	-	37.5% to 50%	86% to 87%	40% to 86%	57.1% to 75%	38.3% to 95%	37.5% to 56%	-	-
Lebanon	-	86.7% to 99%	12% to 94.1%	87.8% to 97.5%	-	17% to 51.2%	35% to 93.3%	4% to 17.7%	63.2% to 85.6%	2.9% to 90%	4.4% to 90%	98.5%
Palestine	99%,	55%	-	71%	32% to 33.3%	-	56% to 66.7%	33.3%	27% to 33.3%	-	20% to 22%	-
Egypt	77% to 100%	60.6% to 94.1%	75% to 98.8%	100%	55%	22.5% to 42.4%	34.4% to 77.6%	18.8% to 32.8%	45% to 52.2%	45.9% to 93.9%	15% to 93.9%	21.2% to 85.8%
Tunisia	100%	72.2%	-	-	-	-	48.5% to 62%	95%	92% to 93%	-	0.8%	-

CN = Gentamycin, CTX = Cefotaxime, AK = Amikacin, IPM = Imipenem, MEM = Meropenem, GAZ = Ceftazidime, CPM = Cefepime, CIP = Ciprofloxacin, TPZ = Piperacillin-tazobactam, ATM = Aztreonam, AUG = amoxycillin-clavulanate, CXM = cefuroxim.

**Table 2:** Increased prevalence for the β-lactamases genes in *E. coli* and ESKAPE pathogens in Arabian region for the years 2000–2020.

Country	Increased β-lactamases Genes Rates during 2000–2020									
	*bla*_SHV_	*bla*_TEM_	*bla*_CTX-M_	*bla*_OXA_	*bla*_PER_	*bla*_GES_	*bla*_VIM_	*bla*_IMP_	*bla*_NDM_	*bla*_VEB_
Saudi Arabia	3.2% to 97.3%	2.6% to 84.1%	8.5% to 87%	26.2% to 85.7%	49.1% to 76.3%	21.7% to 34.5%	7% to 32%	9%	7.4% to 55%	
Emirates	4.4%		21.3%	6.4% & 53.3%					24.7%.	
Qatar	53.2%	40.4%	59% to 66.1%	100%						
Iraq	24%	20% to 46%	26%	58%	17%		5%	9.1% to 50%	67.2%	30%
Lebanon	88.2%	63.2%	41.2% to 86.7%	5.8% to 96.7%		75%	50%	17%	14.7 to 30%	
Palestine	3.3%	22.8% to 33.3%								

**Table 3:** Distribution of total *E. coli* and ESKAPE pathogens in separate Arab region countries during 2000–2020.

Country	*E. coli*	*K. pneumonia*	*P. aeruginosa*	*S. aureus*	*A. baumannii*	*Enterobacter* spp.	Total	Reference
Saudi	22,493	11,959	9854	3829	2582	1316	52,033	[11,12,13,14,15,16,17,18,19,20,21,22,23,24,25,26,27,28,29,30,31,32,33,34,35,36,37]
Emirate	654	524	-	-	-	13	1191	[38,39,40,41,42]
Qatar	448	168	106	-	48	-	770	[43,44,45,46,47,48]
Kuwait	1206	669	79	209	63	7	2233	[49,50,51,52,53,54,55]
Oman	165	112	48	155	107	20	607	[56,57,58,59]
Bahrain	1594	704	60	-	-	62	2420	[60,61,62]
Yemen	130	8	-	-	3	2	143	[63,64,65]
Jordan	316	428	125	-	18	37	924	[66,67,68,69]
Iraq	49	98	196	17	9	13	382	[70,71,72,73,74]
Syria	104	-	-	-	260	-	364	[75,76]
Lebanon	2000	676	40	-	121	-	2837	[77,78,79,80,81]
Palestine	101	15	-	-	-	3	119	[82,83]
Egypt	661	246	356	-	7	19	1289	[84,88,90,91,92,93]
Libya	397	36	-	-	-	-	433	[87,94]
Algeria	232	246	-	106	17	67	668	[95,96,97,98,99]
Tunisia	368	811	210	-	246	15	1650	[100,101,102,104,105]
Morocco	639	113	-	-	-	-	752	[106,107,108,109]
Sudan	450	315	-	54	4	-	823	[110,111,112]
Djibouti	31	-	-	-	-	-	31	[113]
Total	32,038	17,128	11,074	4370	3485	1574	69,669	

**Table 4:** Antibiotic resistance in *E. coli* and ESKAPE pathogens during 2000–2020.

Antibiotic	2000–2005 Mean%	2006–2010 Mean%	2011–2015 Mean%	2016–2020 Mean%
Cefuroxime	44.6	87.2	89.2	87.5
Cefepime	39	47	66.5	73.6
Ceftazidime	49	53.	62.8	72.3
Cefotaxime	62.5	78.0	74.3	72.5
Amoxycillin-Clavulanate	58.6	60	60.6	67.9
Ciprofloxacin	46.8	48.5	59.3	70.3
Amikacin	40.2	47.3	56.9	51.4
Gentamicin	49	50.4	55.2	64.4
Meropenem	30.5		40.2	64.9
Imipenem	30.9		37.6	44.1
Piperacillin-Tazobactam		49	52.7	34.3
Aztreonam	30.6	33.3	73.6	46.5
